# Imagery rescripting and cognitive restructuring for inpatients with moderate and severe depression – a controlled pilot study

**DOI:** 10.1186/s12888-024-05637-y

**Published:** 2024-03-08

**Authors:** Jabin Kanczok, Kamila Jauch-Chara, Franz-Josef Müller

**Affiliations:** 1 Department of Psychiatry and Psychotherapy, University Hospital Schleswig-Holstein, Christian-Albrechts University, Kiel, Germany; 2 Department of Genome Regulation, Max Planck Institute for Molecular Genetics, Berlin, Germany

**Keywords:** Imagery rescripting, Cognitive restructuring, Depression, Fitness wristband

## Abstract

**Background:**

This controlled pilot study investigates the effect of the combined use of cognitive restructuring (CR) and imagery rescripting (IR) compared to treatment as usual among inpatients with moderate and severe depression. Alongside expert ratings and self-report tools, fitness wristbands were used as an assessment tool.

**Methods:**

In addition to the standard inpatient care (SIC) program, 33 inpatients with moderate and severe depression were randomly assigned to an intervention group (two sessions of IR and CR) or an active treatment-as-usual (TAU) control group (two sessions of problem-solving and build-up of positive activity). Depression severity was assessed by the Hamilton Depression Rating Scale-21 (HDRS-21), the Beck Depression Inventory-II (BDI-II), and as a diagnostic adjunct daily step count via the Fitbit Charge 3™. We applied for analyses of HDRS-21 and BDI-II, 2 × 2 repeated-measures analysis of variance (ANOVA), and an asymptotic Wilcoxon test for step count.

**Results:**

The main effect of time on both treatments was η^2^ = .402. Based on the data from the HDRS-21, patients in the intervention group achieved significantly greater improvements over time than the TAU group (η^2^ = .34). The BDI-II data did not demonstrate a significant interaction effect by group (η^2^ = .067). The daily hourly step count for participants of the intervention group was significantly higher (*r* = .67) than the step count for the control group.

**Conclusions:**

The findings support the utilization of imagery-based interventions for treating depression. They also provide insights into using fitness trackers as psychopathological assessment tools for depressed patients.

**Trial registration:**

The trial is registered at the German Clinical Trials Register (Deutsches Register Klinischer Studien) under the registration number: DRKS00030809.

**Supplementary Information:**

The online version contains supplementary material available at 10.1186/s12888-024-05637-y.

## Background

Recent statistics indicate that 280 million people worldwide suffer from depression [21]. Depressive disorders are highly prevalent and take a heavy toll on those who suffer from them. Depression shapes the lives of those affected, their relatives, and, due to absenteeism, their employers and colleagues. Both pharmacological and psychological treatments effectively treat depressive disorders [16].

Despite the efficacy mentioned above, many patients relapse after discontinued pharmacological or psychotherapeutic treatment [25]. Hollon noted a relapse rate of 30.8% in patients who ceased psychotherapy, compared to 76.2% in those who stopped medication treatment. Some relapses may stem from the diminishing effects of treatment, while others occur independently. Psychotherapy, while effective, cannot address every potential stressor. A variety of complex factors, such as comorbid disorders or inadequate social support, also play a significant role in influencing relapse rates*.*

Research into adjunctive therapeutic approaches, alongside the exploration of adjunctive pharmacological or other methods, remains an important objective for the secondary prevention of depressive disorders.

One path of a new adjunctive therapeutic method) addresses the fact that many depressed patients suffer from mental images or memories of painful experiences, which are responsible for a significant portion of their symptoms [8, 11, 33, 42]. Failure to process these mental images can allow them to remain virulent, potentially leading to further suffering [32] as they negatively influence mood [39].

Imagery Rescripting (IR) is a promising technique for addressing psychological complaints associated with unpleasant mental images [31]. Despite its longstanding tradition, dating back to Wolpe and Beck in the 1950s and 1970s, respectively [9, 17], IR has undergone more intensive research in the last decade, revealing encouraging results in the treatment of various mental disorders including post-traumatic stress disorder, social anxiety disorder, body dysmorphic disorder, bulimia nervosa and obsessive compulsive disorder [31].

IR has been applied in recent years mainly to treat patients with PTSD [3, 7, 43]. However, few recent studies have examined the effects of IR patients with depression [10, 32, 41].

Cognitive Restructuring (CR) is another well-established therapeutic technique in which individuals identify, challenge, and replace negative thought patterns to promote more positive and adaptive thinking [38].

Recently, Ma and Lo [30] conducted a study of IR and depression. Their aim was to compare IR with CR (cognitive restructuring: a long-standing technique for treating depression) and to investigate whether the treatments differed in terms of their effectiveness in coping with aversive memories. They found that both depressive symptoms and the number of intrusive memories decreased in both the IR and the CR groups. At follow-up, the IR group’s scores continued to decline, while the CR group’s scores remained at the same level as at post-treatment time. Weaknesses of the study were the one-sided patient population (92% female), the predominant use of self-report instruments, and the relatively low depressiveness of the participants.

To date, CR and IR have not been applied jointly to inpatients with depression. For these reasons, the participants in the present study were treated with both CR and IR. Similar to Averill and colleagues [5], we used fitness bracelets to supplement the diagnostic instruments with activity level as a proxy for functioning and indirectly for mental health.

Previous studies have provided suggestive evidence that an increase in steps count may correlate with changes in activity levels in patients recovering from severe depression [5]. Chia and Zhang demonstrated that passive movement from wearable devices significantly correlated with self-reported and clinician-rated depression severity in individuals with major depressive disorder [13].

In a study of 267 participants, associations were found between depressive symptoms and nighttime heart rate variations and circadian rhythm irregularities. While digital biomarkers had limited ability to detect depression overall, a more accurate model achieved an 80% accuracy in identifying high-risk individuals in specific subsamples [35].

Chan et al. [12] showed that daily running duration, steps per day, and step regularity were identified as independent and significant predictors among participants with incident depressive episodes.

This study is a pilot randomized trial that examines the potential effectiveness of joint use of CR and IR on moderate and severe depression in contrast to a control group. Additionally we examined the feasibility of inpatients wearing a fitness bracelet that provides data as a supplement to self- and expert assessment tools.

We tested the following hypothesis:

Compared to an active psychotherapy group (TAU: treatment-as-usual), the combination of CR and IR is superior in the treatment of depressed inpatients.

## Methods

The study was conducted at the Department for Psychiatry and Psychotherapy at the University Hospital Schleswig–Holstein in Kiel, Germany. Participants with depressive disorders were recruited from the ward. The study was approved by the Ethics Committee of the Medical Faculty at the University of the CAU Kiel (Study ID: D536/19). Recruitment occurred from November 2019 to March 2021.

### Participant recruitment

Participants were women and men (aged 18–76 years) suffering from moderate or severe depressive disorder (either first episode or recurrent episode), as defined by the DSM-5 criteria [4].

In addition, the following parameters had to be met: HDRS-21 > 21 and BDI-II > 19. Participants who met the inclusion criteria were offered an inpatient stay of six- to eight-weeks.

*One participant each from the INT and TAU groups was diagnosed with comorbid PTSD, but no other participants had trauma-related diagnoses. According to Brewin (2010), depressive memories often involve negative life events and interpersonal issues, which were the focus areas in our application of Imagery Rescripting (IR).*

After giving informed consent participants were randomly assigned to either the intervention group (INT) or the treatment-as-usual group (TAU). Outcomes were assessed at the first session (t1, pre-treatment) and at the fourth session (t4, post-treatment). Apart from different psychological interventions (INT/TAU at t2 and t3), all participants received the same standard inpatient care program (SIC). At baseline, all participants received a Fitbit Charge 3™ fitness tracker. All assessments and psychological treatments were conducted by the main author.

### Sample size

Due to the paucity of data on the treatment of IR in depression, a sample size of 40 to 50 was sought as Ma and Lo’ study included 41 subjects [30], and studies on IR and PTSD, and IR and social phobia, included 39 [1] and 23 participants [29], respectively.

### Treatment

The SIC consisted of art/music therapy (60 min per week), as well as physical therapy (25–50 min per week) and relaxation therapy (30–60 min per week), and remained consistent for all participants. Occasionally, there were variations due to absences resulting from illness, but no systematic differences were observed among the participants. The group's accompanied walk followed a uniform format for everyone, with the only divergence being in the custodial outreach, which was tailored to individual needs and could extend up to 25 min per week. Each patient was evaluated for progress and difficulties once a week by the professional staff.

Each week, a medical consultation was provided to determine whether a psychopharmacological treatment was indicated, and if so, medication was offered, which did not include benzodiazepines.

In addition to SIC, all participants received individual psychotherapy sessions once a week for 50 min each. The therapist was a male psychotherapist in training, who had been supervised and trained in IR. After the study, participants continued to receive treatment until remission.

Patients were randomly assigned to the intervention groups by drawing from a drum. Both the allocations of the participants and the implementation of the intervention were carried out by the first author and were not blinded.

#### Intervention group (INT)

The intervention group consisted of two individual therapeutic sessions, held once a week for 50 min. Each session consisted of CR or IR. Participants alternated between CR and IR, starting with one in the initial session and switching to the other in the following session.

#### Cognitive restructuring

CR was conducted using Ellis’ ABC model [18]. The individual steps of the session were as follows:

1. The therapist communicates the cognitive model to the patient.
2. The therapist helps to identify dysfunctional cognitions in a specific problem situation.
3. The therapist challenges instances of dysfunctional cognition.
4. The therapist assists in the development of adequate, functional cognitions.
5. The patient is given homework to practice these new cognitions as new coping skills in problematic situations.

#### Imagery rescripting

Based on Arntz and Weertman [2], and Wheatley and Hackmann [40], the following steps of IR were carried out:

1. The therapist introduces the patient to the idea that memories can be changed and explains why this makes sense.
2. Both search for a distressing memory that is relevant to the current dysfunctional ratings.
3. The therapist asks the patient to imagine the situation in as much detail as possible and to describe it in the present tense.
4. The therapist asks the patient to help their younger self; if they are unable to do so, they are asked to seek help from a) their adult self, b) a fantasy figure, c) another helping person, or d) the therapist (in this order).
5. The therapist instructs the patient to conclude the imaginative exercise with a pleasant outcome of the situation. Special attention is given to meeting the key needs of the younger self.

#### Treatment-As-Usual group (TAU)

The control group also received two separate 50-min individual therapy sessions once a week. *Supportive problem-solving *[27]* was regularly used, often alongside the establishment of positive activities. As per *Hautzinger [23]*, activity protocols and behavioral activation homework were typically introduced in the first or sometimes the second session. Time constraints occasionally limited more in-depth exploration.*

### Measures

In addition to sociodemographic assessments, participants were diagnosed using the DSM-5 criteria for acute, chronic, and recurrent depression (APA, 2022). All assessments were conducted by the first author.

#### Depression symptom measures

##### Main outcome: Third-party assessment

The intensity of depression was assessed using the HDRS-21 [22], a clinician-administered assessment of psychological and somatic symptoms of depression and their severity. The patient responds to 21 items about his or her complaints, and these responses are translated into a total score. The total score was used for subsequent analysis.

##### Secondary outcome: Self-assessment

The BDI-II is the most widely used self-report measure of depression [28]. It consists of 21 items that represent the majority of the DSM-IV criteria for an episode of major depression, during the previous two weeks. Subica et al. supported the BDI-II as a screening instrument for overall depressive symptomology and as a measure of clinical changes to depressive symptomology over time. But since their ROC analyses showed poor sensitivity/specificity balance they raise awareness for the limitations of the BDI-II as a diagnostic tool for adult inpatients (2014). Following Subica et al. we concluded to choose the HDRS-21 as the primary outcome measure and the BDI-II as a secondary outcome. In clinical settings where clinician judgment is readily available, the HDRS-21 may provide additional insights.

##### Secondary outcome: Activity level

The wearer's daily/hourly step count was measured using the FitBit Charge 3™ fitness wristband. For processing of the raw step count data, the wristband data were pseudonymized and uploaded to Fitabase (https://www.fitabase.com).

#### Statistical methods

Data analysis was performed using SPSS 27. Descriptive statistics, mean values, standard deviation, median, and interquartile range were calculated.

To determine whether there was a difference between the two groups on the main outcome (HDRS-21) and the secondary outcomes (BDI-II, daily step count), a mixed ANOVA was carried out in each case. A 2 (time; t1 vs. t4) × 2 (condition; TAU vs. INT) repeated measures ANOVA was used to test for the effectiveness of the treatments. The η^2^ was calculated to measure effect size.

Daily step counts < 500 were removed from the data prior to the activity analysis as this would have been the case of incorrect measurements. Participants who accumulated more steps than 1.5 times the standard deviation of all participants were also removed as outliers. The time window from Monday to Friday, 7 am to 9 pm, was considered. The Kolmogorv-Smirnov test and the Shapiro–Wilk test were performed to test the normal distribution. The significance level was set at 0.05. To depict the progression of step counts for both the intervention and control groups, a LOESS smoothing curve was applied. A Pearson correlation was computed to explore the potential relationship between an improved HDRS-21 score and an increase in daily steps taken.

## Results

### Participants

**Fig. 1:**
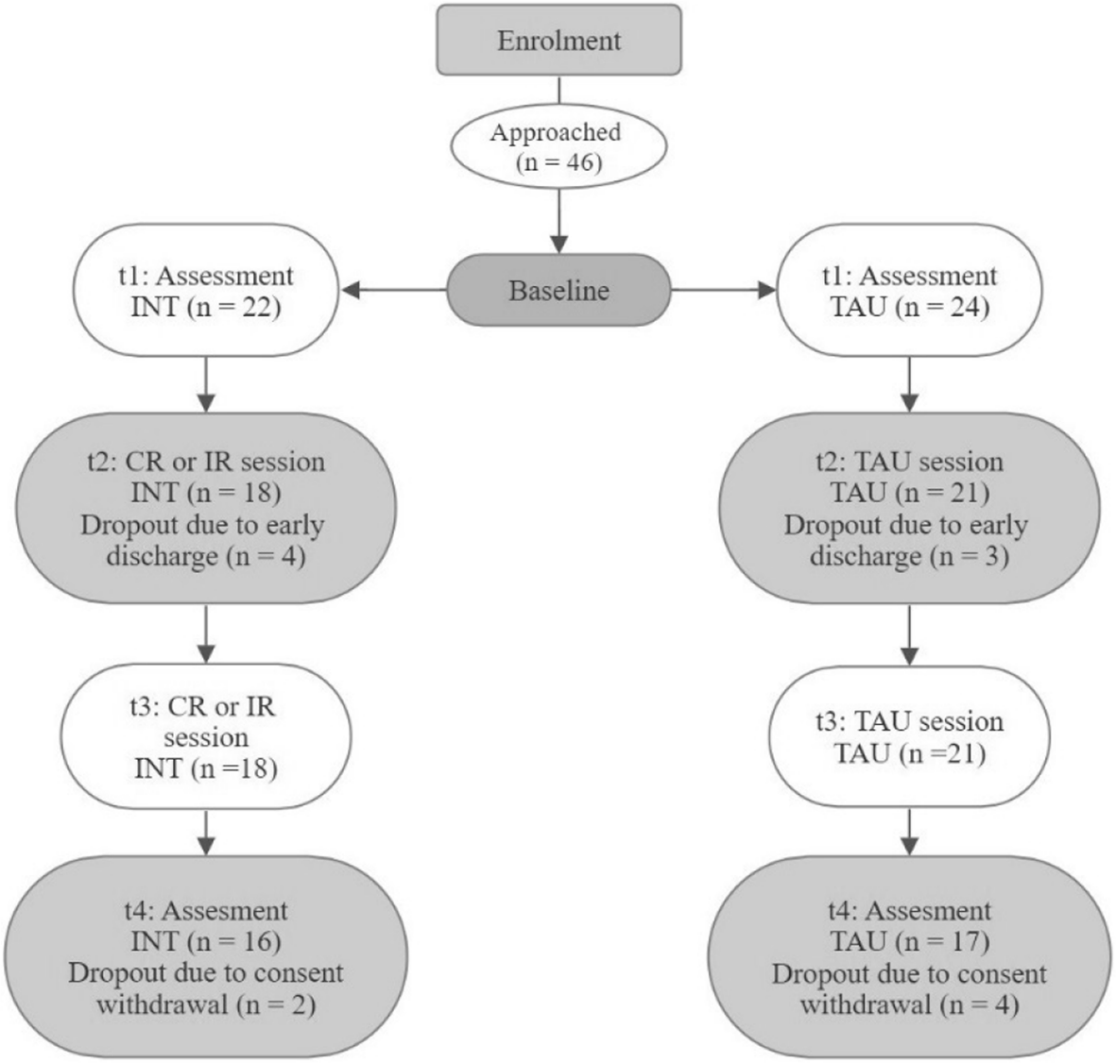
Study flow chart

The final sample consisted of 46 participants assigned to the two treatment conditions (INT: *n* = 16; TAU: *n* = 17; for dropouts, see flow chart in Fig. 1). Thirteen participants were excluded due to early voluntary discharge from the inpatient unit or lack withdrawal of informed consent. The INT group consisted of 8 women and 8 men, with a mean age of 39.1 (*SD* = 14.1); 50% had recurrent major depression, and 68.75% had a comorbid disorder (personality disorder, substance use disorder, or anxiety disorder).

The TAU group consisted of 13 women and 4 men, with a mean age of 45.1 years (*SD* = 14.7). 52.9% had recurrent major depression, and 35.3% had a comorbid disorder (personality disorder, substance use disorder, or anxiety disorder). Table 1 provides a summary of the sample and demographic characteristics at baseline. Initially, the two groups showed no significant differences in HDRS-21 and BDI-II scores or in daily step counts.

**Table 1:** Baseline sample characteristics and measure scores of the treatment groups

	INT (*n* = *16*)	TAU (*n* = *17*)	Test statistic	***p***-value
Female (*n (%)*)	8 (50)	13 (76.5)	X^2^ = 2.496	.114
Age (*M (SD)*)	39.1 (14.1)	45.1 (14.7)	*t* = -1.17	.251
Comorbidity (*n (%)*)	11 (68.8)	6 (35.3)	X^2^ = 3.694	.055
Anxiety disorders (*n (%)*)	4 (25)	2(11.8)		
Personality disorders (*n (%)*)	3(18.8)	3(17.6)		
Measures				
HDRS-21 (M(SD))	31.31 (5.1)	28.12 (6.5)	*t* = 1.56	.129
BDI-II (M(SD))	32.81 (10.2)	30.82 (10.4)	*t* = .556	.582
daily steps (M(SD))	9449 (3786)	7182 (2851)	*t* = 1.807	.081

The baseline daily steps consists of the mean of days 1 to 5

**Fig. 2:**
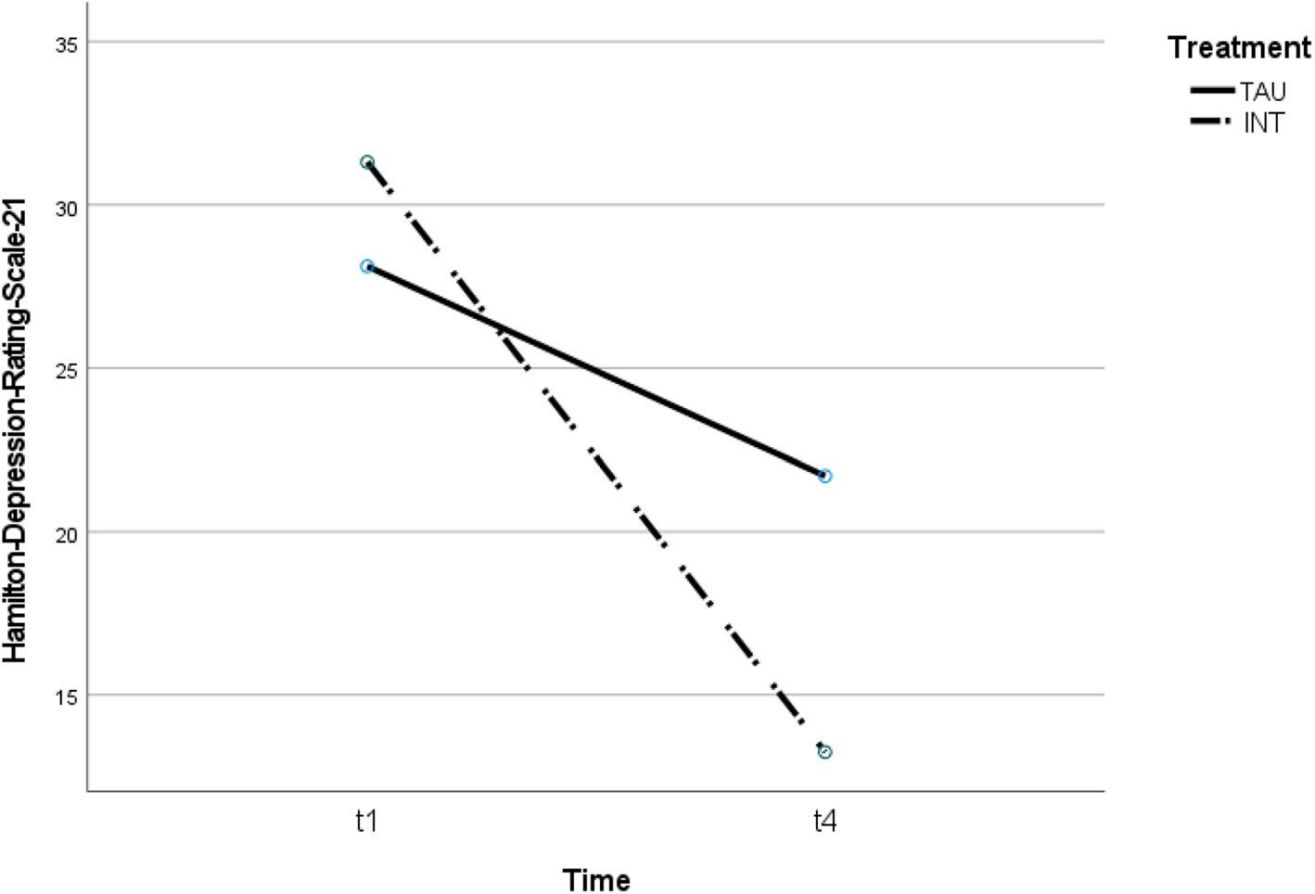
Treatment effect of intervention (INT) and treatment-as-usual (TAU) on depression (expert rating)

**Fig. 3:**
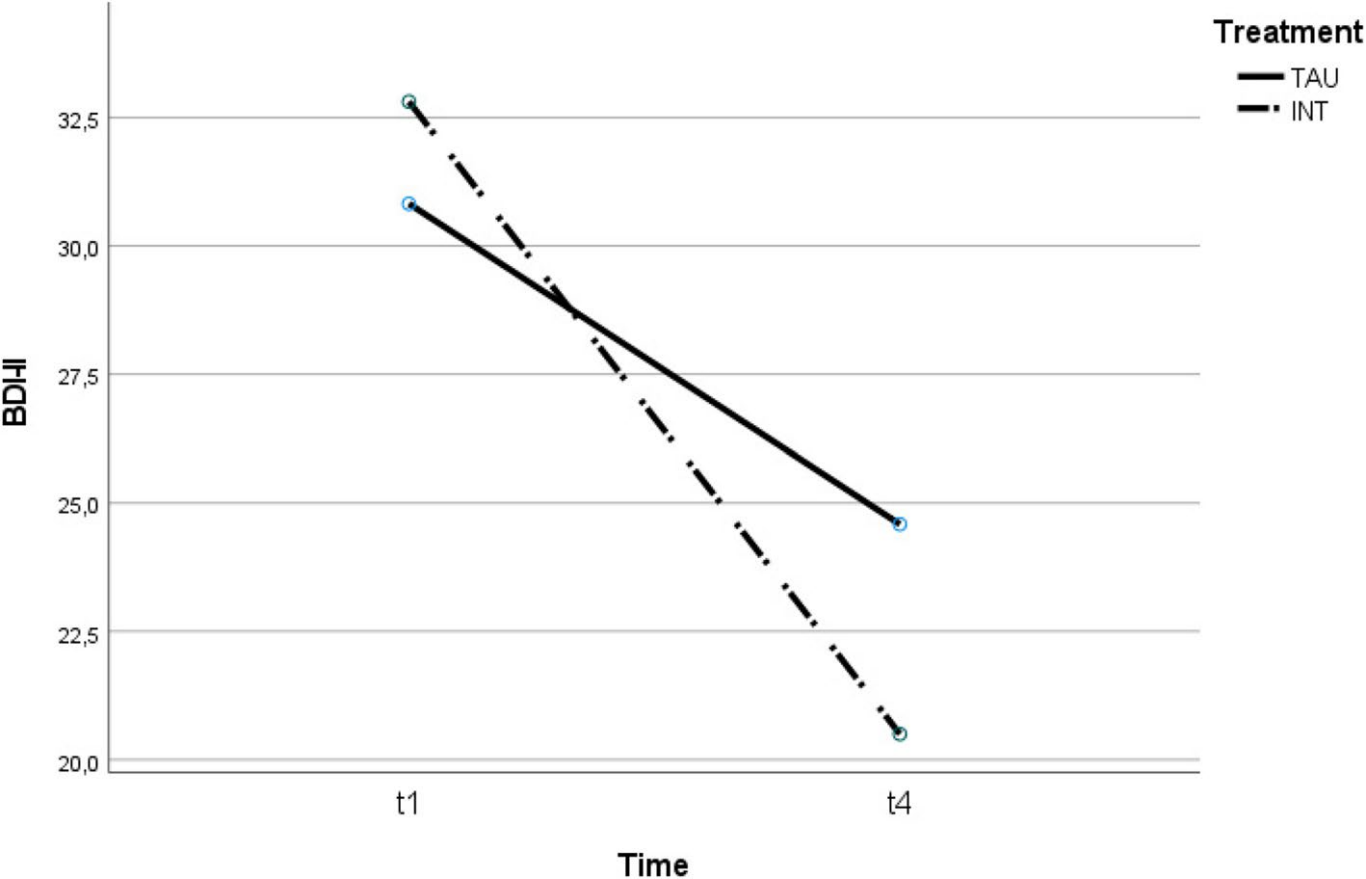
Treatment effect of intervention (INT) and treatment-as-usual (TAU) on depression (self-report rating)

See Table 2 for a summary of the mean scores and Figs. 2 and 3 for the trajectory of change for each outcome measure at the two time points.

**Table 2:** Means, standard deviations, and effect sizes (Cohen’s *d*) for the change pre- and post-treatment

	Pre-treatment	Post-treatment	
	M (SD)	M (SD)	d
HDRS-21			
INT	31.31 (5.1)	13.25 (7.7)	2.77
TAU	28.12 (6.5)	21.71 (8.6)	.84
BDI-II			
INT	32.81 (10.2)	20.50 (12.9)	1.06
TAU	30.82 (10.4)	24.59 (12.4)	.54
Daily steps			
INT	9450 (3787)	10,861 (3232)	-.40
TAU	7183 (2852)	9019 (3805)	-.55

*HDRS-21* Hamilton Depression Rating Scale-21, *BDI-II* Beck Depression Inventory-Second Edition; daily steps = pre (mean of days 1–5), post (mean of days 15–20)

### Primary outcome: Hamilton Depression Rating Scale-21 (HDRS-21)

There was no main effect of group (*F*(1,31) = 2.44, *p* = 0.13), indicating that the two treatment groups were not significantly different at baseline. There was a main effect of time (*F*(1,31) = 71.89, *p* < 0.001, ῃ^2^ = 0.69), demonstrating a significant reduction in depression scores over time.

In addition, there was a significant interaction effect, indicating that the INT group achieved significantly greater improvements than the TAU group (*F*(1.31) = 16.29, *p* < 0.001, ῃ^2^ = 0.34). Figure 2 shows the overall positive treatment effect of the applied treatment on depression.

### Secondary outcome: Beck-Depression-Inventory-II (BDI-II)

There was no main effect of group (*F*(1,31) = 0.309, *p* = 0.58), indicating that the two treatment groups were not significantly different at baseline. There was a main effect of time (*F*(1,31) = 20.88, *p* < 0.001, ῃ^2^ = 0.40), demonstrating a significant reduction in depression scores over time. There was no statistically significant interaction between time and group (*F*(1,31) = 2.24, *p* = 0.144, ῃ^2^ = 0.067). Figure 3 shows the trajectory of change in the given self-report rating.

### Secondary outcome: activity level employing daily hourly step count

**Fig. 4:**
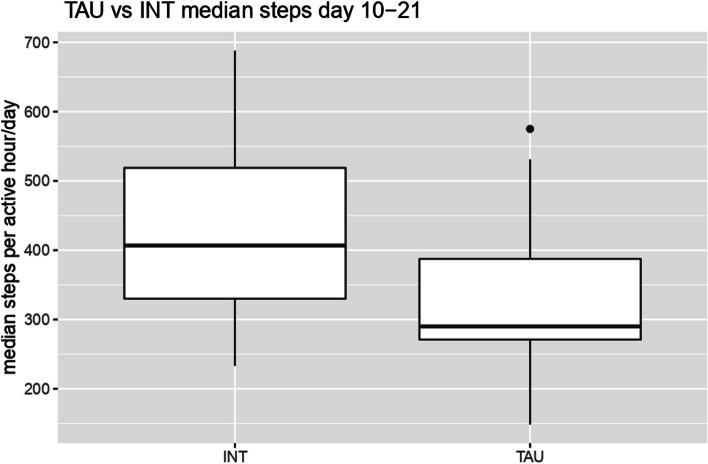
Hourly step count of the pooled intervention group and control group after the first intervention. Adjusted for day and time of the week. Legend: TAU: Treatment-as-Usual-Group; INT: Intervention-Group. Active hour per day includes steps taken between Mon–Fri from 7 am to 9 pm

**Fig. 5:**
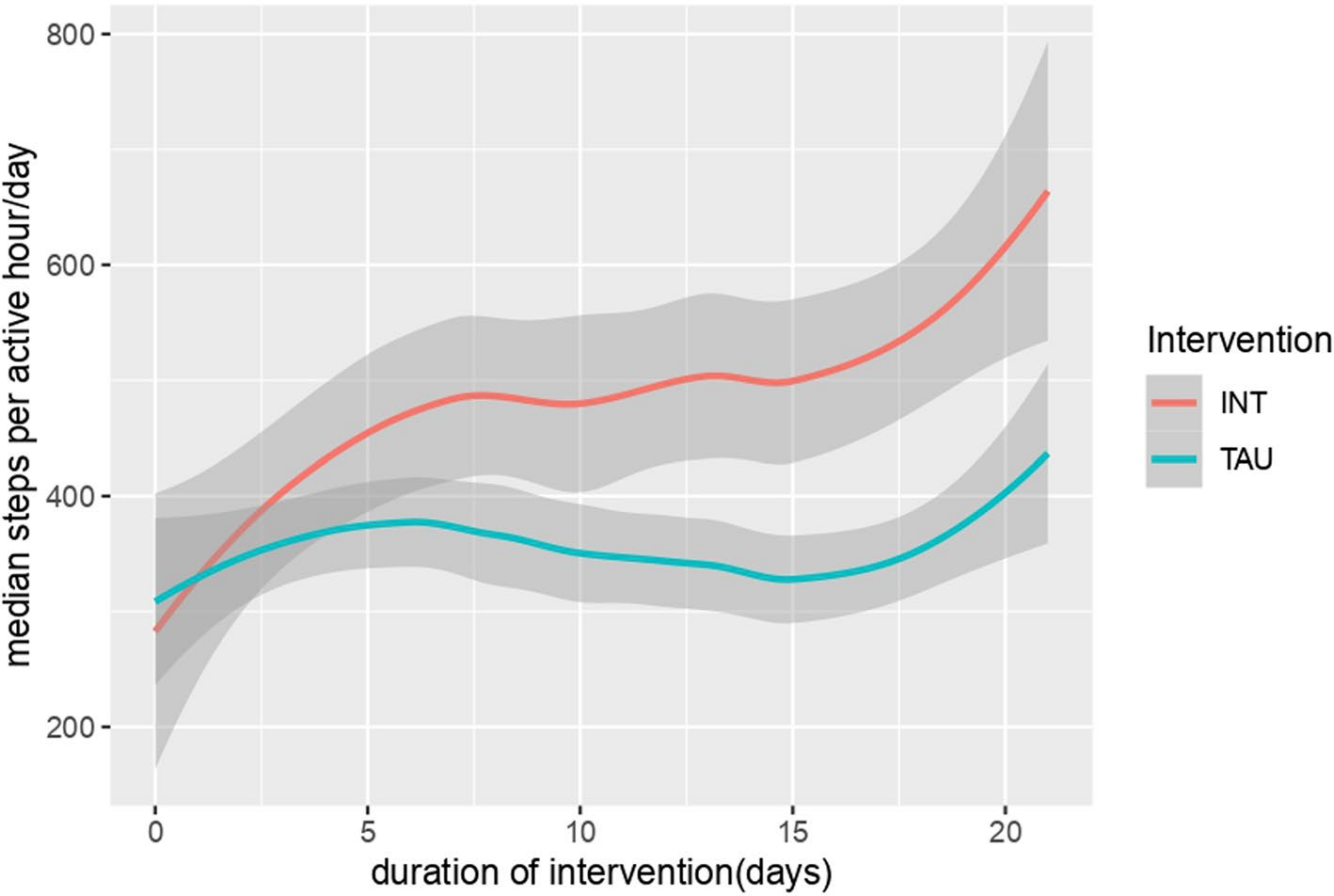
Hourly step count of the pooled intervention group and control group from the start to the end of the study. Adjusted for day and time of the week. Legend: TAU: Treatment-as-Usual-Group; INT: Intervention-Group. Active hour per day includes steps taken between Mon–Fri from 7 am to 9 pm

There was no main effect of group (*F*(1,10) = 0.39, *p* = 0.55), indicating that the two treatment groups were not significantly different at baseline. The daily hourly step count (Mon–Fri from 7 am to 9 pm) was significantly higher after the intervention (days 10 to 21) (Mdn = 394.50) than before (Mdn = 283.00; asymptotic Wilcoxon test: *z* = -3,733, *p* = 0.01157, *n* = 31), with an effect size of *r* = 0.67. This is shown in Fig. 4. Figure 5 shows the progression of step count for the intervention group and control groups over the entire study period. Furthermore, we found a non-significant positive correlation (*r* = 0.12, *p* = 0.49) between improved HDRS-21 scores and daily steps taken, suggesting no statistically significant relationship.

## Discussion

The present study suggests that the combined use of CR and IR may show promise in the treatment of moderate and severe depression. However, it is important to note the presence of several limitations, for example a relatively small sample size, and the absence of manualization/standardization of the procedure. We were also able to show that the use of fitness wristbands is a feasible method for assessing the activity levels of depressed inpatients. In our study, we examined the relationship between HDRS-21 score improvements and daily step counts. The Pearson correlation showed a non-significant positive correlation (*r* = 0.12, *p* = 0.49), suggesting no statistically significant link between HDRS-21 improvements and step count. Despite this, the positive correlation hints at a possible trend, though it should be interpreted cautiously due to numerous missing data points. This highlights the necessity for further research with more complete data to confirm and expand upon these results.

In this study, depressed patients benefited from CR, IR, and TAU on all measures (clinician and self-report and daily step count). In the clinician-administered assessment using the HDRS-21, the present study shows that psychotherapeutic treatment with IR and CR is more effective than supportive psychotherapy focused on problem solving (TAU). Patients in the intervention group were significantly (*p* < 0.001) less depressed than patients in the control group. The effect size was η^2^ = 0.34, which is considered a small effect. Recently, Hengartner and Plöderl [24] noted that statistical significance does not imply clinical significance or relevance. It has been shown that patients perceive a beneficial effect if the HDRS-21 score is 2.8 to 4.6 points lower after the intervention [34]. In the intervention group, all but one participant reduced their score by more than 5 points (93.75%). In the control group, 83.4% scored 3 to 5 points lower than before the intervention. For the BDI-II, the clinically relevant minimally important difference is between 3 and 6 points [24]. In the intervention group, all but two participants reduced their scores by 3 or more points. In the control group, 76.5% scored 3 or more points lower than before the intervention. Regarding the self-assessment tool BDI-II, no significant interaction effect could be detected in the intervention group, although the group effect is small: η^2^ = 0.067. Subica et al. [37] found that the BDI-II has limited sensitivity/specificity in adult inpatients with Major Depressive Disorder, possibly explaining its inability to detect an interaction effect in our study. The BDI-II might not be sensitive enough to capture changes from the intervention. Therefore, we selected HDRS-21 as the primary outcome due to its suitability for detecting specific intervention outcomes. Additionally, the BDI-II's focus on cognitive items, which typically take longer to change [20], might also contribute to the absence of an interaction effect.

Previously, Ma and Lo [30] compared treatment with CR to treatment with IR in depressed patients. Depressive symptoms were reduced in both groups. However, the duration of the treatment sessions were 10 to 40 min longer than in the current study. Our study, which used a combined approach of IR, CR, and standard intervention, supports Ma and Lo's findings by suggesting that this integrated method is effective in treating depression, even though our focus was not solely on IR as a standalone intervention. The main effect of time (η^2^ = 0.402) suggests an overall effectiveness of both treatments. Our result is consistent with the main finding of the clinician-administered assessment, as well as with the results of Ma and Lo [30], who additionally reported a significant interaction effect in their BDI-II results. The fact that the effect in our study is similar to that in the study by Ma and Lo but not significant may be due to Ma and Lo’s greater emphasis on homework between sessions and treatment of less impaired study participants [30]. As in the study by Ma and Lo, we were unable to obtain a representative sample in terms of gender. Our control group has an above-average number of females. A gender balance was not achieved, as the underlying population (inpatients in our ward) was predominantly female.

The fitness wristbands data showed that the daily hourly step count (Mon–Fri from 7 am to 9 pm) was significantly higher after the intervention (days 10 to 21) than before (days 1 to 5). The effect size (*r* = 0.67) is consistent with a strong effect according to Cohen [15].

The fitness wristband results are corroborated by both the HDRS-21 and the BDI-II measurements and show that patients become more active after treatment with IR and CR. These results, in line with previous studies [5, 14, 26] are suggestive that a reduction in depressive symptoms is correlated with an increased activity level, which in turn is quantifiable with commercial fitness tracker systems.

However, there are several limitations to our study, for example:

*We addressed a key challenge in the INT group where a participant's comorbid PTSD diagnosis risked skewing the Imagery Rescripting (IR) focus towards trauma instead of depression. To avoid misinterpreting improvements, we strictly confined the IR content to classical depressive themes, particularly negative life events and interpersonal issues, thereby reducing potential bias.*

A weakness of our study is that the same therapist treated both groups and administered the HDRS-21 assessments. Although this should not affect the self-report or fitness wristband measures, it could introduce a bias in treatment, despite the supervision by a physician and continuous professional exchange in patient-centered team meetings.

Another limitation is the small final sample size of 33 analyzed participants (despite our target of 40–50). Retrospective calculation (G*Power; [19] showed that at least 54 participants would have been needed to reach a significant effect of η2 = 0.25 between intervention groups on the BDI-II self-report measure.

In addition, the interpretation of fitness wristband-based measures is not straightforward. Activity data could only be interpreted during the hours of 7 am to 9 pm, Monday through Friday. Without this restriction to a defined time window during which most daily activities occur on an inpatient unit, the data were too erratic or too skewed to satisfactorily apply inferential statistical tests.

Moreover, there are several pitfalls in using daily step counts to infer activity levels and, by proxy, higher or lower levels of depression. For example, it is conceivable that patients might accumulate more steps because they suffer from agitated depression and cannot come to rest. Alternatively, depressed patients might take more steps in an inpatient setting because they might have to attend increasingly more appointments with e.g. social workers, group therapy or physical therapy over the length of their stay. An increased step count does not necessarily imply reduced depression. Our correlation analysis showed no significant link between depression and step count, but a more comprehensive study might reveal a relationship between these variables. Future studies using similar methods could include, e.g., questionnaires helping to better understand confounding factors that may further increased step counts. Due to the measures taken throughout the coronavirus pandemic, some patients were unable to leave the inpatient ward for longer periods for weeks on end, which also may have biased the step count data.

The number of steps and the inferences drawn about decreased energy and activity represent only a small subset of depressive symptoms.

Sleep is also highly relevant in depressed patients as central part the symptom complex. However, we found that sleep could not be reliably measured by the Fitbit Charge 3™ wristbands employed in this study with depressed patients. For tracking sleep, Berryhill et al. [6] have reported that the WHOOP Strap 2.0™ fitness wristband could track sleep almost as accurately as laboratory polysomnography. Importantly, however, all these technologies and especially the underlying inference algorithms have only been validated in heathy subjects and may produce uninterpretable results in subjects with severely disrupted sleep architectures.

In essence, however, the increasingly perfected technologies and algorithms underlying fitness wristbands can significantly expand the validity and range of assessments of depression.

An additional limitation is the significant variability in medication among individuals and adjustments made during their inpatient stay. These varying medication regimens could introduce confounding factors, necessitating a detailed examination in future research. Moreover, we note that the standard program of care was largely uniform across participants, with differences primarily in the level of assistance sought from custodians.

For future studies, it is important to investigate whether any differences between IR and CR can be also found in a representative sample (i.e., a balanced number of male and female participants). In China, Ma and Lo [30] have already demonstrated this in a partially representative sample (92% of participants were female). Studies have shown that the odds ratio for gender differences in major depression diagnoses is 1.95 [36]. This translates to almost twice as many women experiencing major depression. For future studies to be representative of the population, the ratio of women to men should be closer to 2:1. Otherwise, the results may not generalize to the population at large.

Future research could explore the effectiveness of IR by gathering patient reports on their emotional state, distress intensity from thoughts or images, and overall well-being changes. A decrease in reported emotional distress, anxiety, or symptoms linked to targeted mental images could suggest a positive intervention impact. Moreover, a 100 mm visual analog scale (VAS) or digital representation displayed in an application on a smartphone could be used to assess participants' emotions before and after IR sessions.

Our study could help to enrich the empirical body of work available on IR in depression. Our findings suggest that using a combination of IR and CR may reduce depression symptoms. However, given the small sample size and the nature of the combined intervention, further investigation into IR's role in treating depression is necessary. Our study's limitations, including its sample size and the constraints of the combined approach, mean that while our results hint at IR's potential benefits, more extensive and controlled research is essential before recommending IR as a standard treatment for depression.

Notwithstanding its limitations, our study offers key preliminary insights, highlighting IR and CR as promising approaches in treating depression. It sets the stage for future, more in-depth research that may refine therapeutic methods for depression.

### Supplementary Information

**Supplementary Material 1.**

## Data Availability

The dataset generated and analyzed in the current study can be obtained in an anonymized format from the corresponding author upon reasonable request.
