# Supplementary material for: Imagery rescripting and cognitive restructuring for inpatients with moderate and severe depression – a controlled pilot study
Source: BMC Psychiatry. 2024 Mar 8;24:194. doi: 10.1186/s12888-024-05637-y (PMC10921678; doi:10.1186/s12888-024-05637-y)
Supplement: Supplementary file 1 — Supplementary Material 1. [file 12888_2024_5637_MOESM1_ESM.docx]

Data Import and plotting for Fitbit data

This Notebook provides functions and code for explorative analysis of Fitbit data. The Data is asumed to exist in a folder with the following structure:

1. A CSV file containing start and stop dates, Patient ID and important clincal covariates.
2. Daily sleep summary files named following ‘Patient_ID_dailySteps_…’ pattern
3. Daily activity summary files named following ‘Patient_ID_sleepStagesDay_’.
4. High temporal Resolution sleep data named following ‘Patient_ID_30secondSleepStages’ .
5. High temporal Resultion activity data named following the ’Patient_ID__hourlySteps’ pattern.

It is asumed that there is only one such file per patientht and that multiple Files stemming from separate exports are merged.

Time and date are handled with the *lubridate* package which is localisation and language setting sensitive.I.e.: “Monday, Tuesday” or “Montag, Dienstag”.

The aim of this analysis is to establish the data quality and characteristics which can be expected from Activity tracking in a clincal setting.

# Data Summary

All but one Patient consented to wear the Activity tracker. Leaving 17 Patients in the Control treatment group (TAU) and 15 in the combinded CR+IR treatment group.

One additional Patient had no reported data in file P510020

CR IR TAU 7 7 17

# Initialisation

We first read the overview file which contains PatientIDs, Quaracteristics and study dates.

require(ggplot2)

## Loading required package: ggplot2

## Warning: package 'ggplot2' was built under R version 4.0.4

require(lubridate)

## Loading required package: lubridate

## Warning: package 'lubridate' was built under R version 4.0.5

##
## Attaching package: 'lubridate'

## The following objects are masked from 'package:base':
##
## date, intersect, setdiff, union

Uebersicht<-read.csv("Daten_JK/Uebersicht2.csv")
Uebersicht$Intervention<- rep("TAU",nrow(Uebersicht))
Uebersicht$Intervention[Uebersicht$IV..TAU.!="TAU"]<-"INT"
Uebersicht$HAMD17_DIFF<- Uebersicht$Prä.HAMD17- Uebersicht$Post.HAMD17
head(Uebersicht)

## neue.Einw. Trackergerät Trackerpw Trackeraccount
## 1 x 27 doktorarbeit123 p5studieka+0273@gmail.com
## 2 x 22 doktorarbeit p5studieka+0223@gmail.com
## 3 x 29 doktorarbeit123 p5studieka+0293@gmail.com
## 4 x 22 doktorarbeit123 p5studieka+0226@gmail.com
## 5 Mitte Juni 24 doktorarbeit123 p5studieka+0244@gmail.com
## 6 x 26 doktorarbeit123 p5studieka+0261@gmail.com
## Probandennummer Einschlussdatum IV..TAU. X X1..EG Prä.BDI Prä.HAMD.21
## 1 P510022 19.08.2020 CR NA 19.08.2020 20 25
## 2 P510023 19.08.2020 CR NA 19.08.2020 20 21
## 3 P510024 19.08.2020 CR NA 19.08.2020 38 33
## 4 P510032 04.11.2020 CR NA 04.11.2020 36 35
## 5 P510033 16.11.2020 CR NA 16.11.2020 30 34
## 6 P510036 02.12.2020 CR NA 02.12.2020 21 33
## Post.BDI Post.HAMD.21 X2..EG X3.EG X4.EG..Ende TAU CR IR Prä.HAMD17
## 1 7 3 27.08.2020 03.09.2020 09.09.2020 NA 1 NA 19
## 2 6 4 26.08.2020 02.09.2020 04.09.2020 NA 1 NA 20
## 3 34 22 28.08.2020 03.09.2020 09.09.2020 NA 1 NA 29
## 4 16 11 11.11.2020 18.11.2020 25.11.2020 NA 1 NA 31
## 5 40 16 23.11.2020 04.12.2020 10.12.2020 NA 1 NA 31
## 6 17 9 09.12.2020 16.12.2020 23.12.2020 NA 1 NA 29
## Post.HAMD17 leichte.Depr Schwere.Dep Intervention HAMD17_DIFF
## 1 3 1 NA INT 16
## 2 4 1 NA INT 16
## 3 18 NA NA INT 11
## 4 8 1 NA INT 23
## 5 13 NA NA INT 18
## 6 8 1 NA INT 21

Treat<-Uebersicht$Intervention
names(Treat)<-Uebersicht$Probandennummer
Hamd17Group<-rep("M",nrow(Uebersicht))
names(Hamd17Group)<- Uebersicht$Probandennummer
Hamd17Group[Uebersicht$Post.HAMD17>=21]<-"S"
Hamd17Group[Uebersicht$Post.HAMD17<=11]<-"L"

# First look

Treatment as usual (TAU) and Intervention group have similar starting HAMD17 scores.


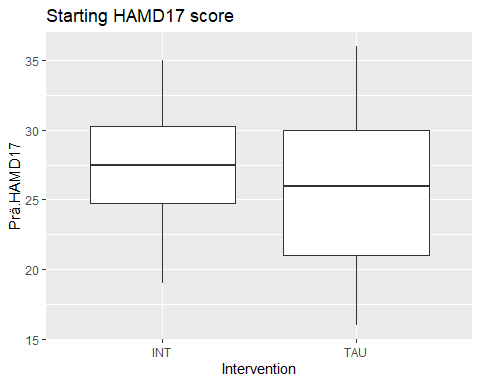


End of Intervention HAMD17 scores improved for the treatment group.

g<-ggplot(Uebersicht,aes(y=Post.HAMD17,x=Intervention))+geom_boxplot()+labs(title="End HAMD17 score")
print(g)


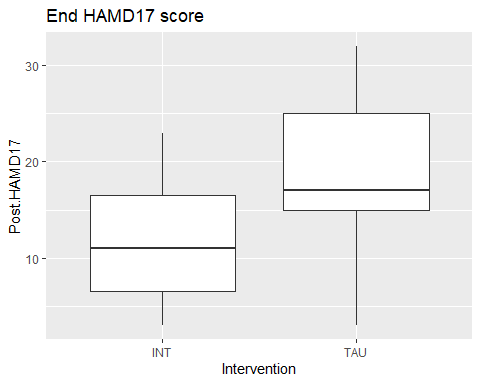


g<-ggplot(Uebersicht,aes(y=HAMD17_DIFF,x=Intervention))+geom_boxplot()+labs(title="Improvement in HAMD17 scores")
print(g)


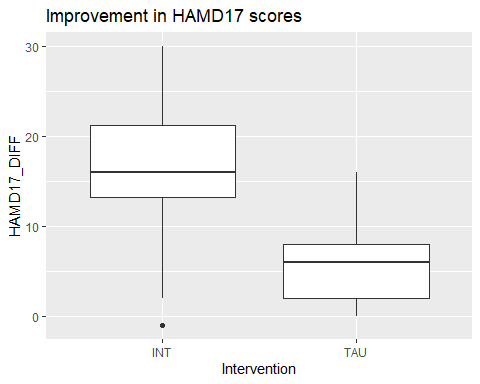


# Steps

Individual data sets are read through accessory functions which take patientID, start and a stop dates.

readDailySteps<-function(Patient,start,stop){
 f<-list.files("./Daten_JK/",pattern = paste(Patient,"_dailySteps_",sep="",collapse = ""),full.names = T)
 #dmy("20.08.2020")
 #print(f)
 print(f)
 StepsCSV<-read.csv(f[1])
 TimePoints<-parse_date_time(StepsCSV[,1],'%m/%d/%Y')
 StepsCSV["TimePoints"]<-TimePoints
 StepsCSV[,"Weekdays"] <- weekdays(TimePoints)
 selTime<- TimePoints >=dmy(start) & TimePoints<=dmy(stop)
StepsCSV$fromStart<-as.numeric(date(TimePoints)-dmy(start))
StepsCSV$toEnd<-as.numeric(date(TimePoints)-dmy(stop))
StepsCSV$PatientID<-Patient
StepsCSV[selTime,]
}

For instance we can access and store the daily steps the patient “P510033” who started “16.11.2020” and ended “16.11.2020” by running:

i=5 # "P510033
P= Uebersicht[i,"Probandennummer"] # "P510033"
S<- Uebersicht[i,"X1..EG"] #"16.11.2020"
E<-Uebersicht[i,"X4.EG..Ende"] #"16.11.2020"
DF<-readDailySteps(P,S ,E)

## [1] "./Daten_JK/P510033_dailySteps_20191020_20210526.csv"

head(DF)

## ActivityDay StepTotal TimePoints Weekdays fromStart toEnd PatientID
## 1 11/16/2020 3802 2020-11-16 Montag 0 -24 P510033
## 2 11/17/2020 9331 2020-11-17 Dienstag 1 -23 P510033
## 3 11/18/2020 8756 2020-11-18 Mittwoch 2 -22 P510033
## 4 11/19/2020 6556 2020-11-19 Donnerstag 3 -21 P510033
## 5 11/20/2020 11435 2020-11-20 Freitag 4 -20 P510033
## 6 11/21/2020 5124 2020-11-21 Samstag 5 -19 P510033

## Concatenate

for(i in 1:33){

 P<- Uebersicht[i,"Probandennummer"]
 if(P=="P510040") next
 S<- Uebersicht[i,"X1..EG"]
 E<-Uebersicht[i,"X4.EG..Ende"]
 DF<-readDailySteps(P,S,E)
 if(nrow(DF)==0) next
 DF$Intervention<- Uebersicht$Intervention[i]
 DF$Post.HAMD<-Uebersicht$Post.HAMD17[i]
 DF$Prä.HAMD<-Uebersicht$Prä.HAMD17[i]
 DF$Post.BDI<-Uebersicht$Post.BDI[i]
 DF$Prä.BDI<-Uebersicht$Prä.BDI[i]
 DF$Post.HAMD.GROUP<-"M"
 if (Uebersicht$Post.HAMD17[i]<= 11) DF$Post.HAMD.GROUP<-"L"
 if (Uebersicht$Post.HAMD17[i]>= 21) DF$Post.HAMD.GROUP<-"S"
 DF$Prä.HAMD.GROUP<-"M"
 if (Uebersicht$Prä.HAMD17[i]<= 11) DF$Prä.HAMD.GROUP<-"L"
 if (Uebersicht$Prä.HAMD17[i]>= 21) DF$Prä.HAMD.GROUP<-"S"


 if(i ==1) DFALL<-DF else DFALL<-rbind(DFALL,DF)
}

## [1] "./Daten_JK/P510022_dailySteps_20191020_20210526.csv"
## [1] "./Daten_JK/P510023_dailySteps_20191020_20210526.csv"
## [1] "./Daten_JK/P510024_dailySteps_20191020_20210526.csv"
## [1] "./Daten_JK/P510032_dailySteps_20191020_20210526.csv"
## [1] "./Daten_JK/P510033_dailySteps_20191020_20210526.csv"
## [1] "./Daten_JK/P510036_dailySteps_20191020_20210526.csv"
## [1] "./Daten_JK/P510042_dailySteps_20191020_20210526.csv"
## [1] "./Daten_JK/P510001_dailySteps_20191020_20210526.csv"
## [1] "./Daten_JK/P510002_dailySteps_20191020_20210526.csv"
## [1] "./Daten_JK/P510007_dailySteps_20191020_20210526.csv"
## [1] "./Daten_JK/P510011_dailySteps_20191020_20210526.csv"
## [1] "./Daten_JK/P510014_dailySteps_20191020_20210526.csv"
## [1] "./Daten_JK/P510017_dailySteps_20191020_20210526.csv"
## [1] "./Daten_JK/P510018_dailySteps_20191020_20210526.csv"
## [1] "./Daten_JK/P510021_dailySteps_20191020_20210526.csv"
## [1] "./Daten_JK/P510025_dailySteps_20191020_20210526.csv"
## [1] "./Daten_JK/P510026_dailySteps_20191020_20210526.csv"
## [1] "./Daten_JK/P510030_dailySteps_20191020_20210526.csv"
## [1] "./Daten_JK/P510041_dailySteps_20191020_20210526.csv"
## [1] "./Daten_JK/P510043_dailySteps_20191020_20210526.csv"
## [1] "./Daten_JK/P510044_dailySteps_20191020_20210526.csv"
## [1] "./Daten_JK/P510045_dailySteps_20191020_20210526.csv"
## [1] "./Daten_JK/P510046_dailySteps_20191020_20210526.csv"
## [1] "./Daten_JK/P510047_dailySteps_20191020_20210526.csv"
## [1] "./Daten_JK/P510005_dailySteps_20191020_20210526.csv"
## [1] "./Daten_JK/P510006_dailySteps_20191020_20210526.csv"
## [1] "./Daten_JK/P510008_dailySteps_20191020_20210526.csv"
## [1] "./Daten_JK/P510009_dailySteps_20191020_20210526.csv"
## [1] "./Daten_JK/P510020_dailySteps_20191020_20210526.csv"
## [1] "./Daten_JK/P510037_dailySteps_20191020_20210526.csv"
## [1] "./Daten_JK/P510038_dailySteps_20191020_20210526.csv"
## [1] "./Daten_JK/P510039_dailySteps_20191020_20210526.csv"

# Compare Steps

Early data does not show difference between intervention and TAU.

sel1<- DFALL$fromStart<10 & DFALL$Weekdays%in%c("Montag","Dienstag","Mittwoch","Donnerstag","Freitag")
tt<-tapply(DFALL$StepTotal[sel1],DFALL$PatientID[sel1],mean)
DF1<-data.frame(MeanStepsDay=tt,Treatment=Treat[names(tt)])
g1<-ggplot(DF1,aes(y=MeanStepsDay,x=Treatment) )+geom_boxplot() +labs(title="mean daily steps days 0-9")
print(g1)


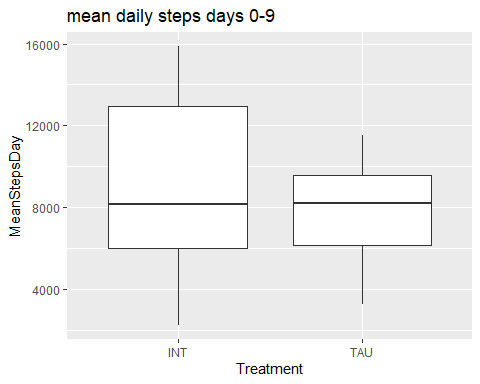


wilcox.test(tt[Treat[names(tt)]=="TAU"],tt[Treat[names(tt)]!="TAU"] )

##
## Wilcoxon rank sum exact test
##
## data: tt[Treat[names(tt)] == "TAU"] and tt[Treat[names(tt)] != "TAU"]
## W = 107, p-value = 0.6526
## alternative hypothesis: true location shift is not equal to 0

table(Treat[names(tt)]=="TAU")

##
## FALSE TRUE
## 14 17

median(tt[Treat[names(tt)]=="TAU"])

## [1] 8199.875

median(tt[Treat[names(tt)]!="TAU"])

## [1] 8168.845

IQR(tt[Treat[names(tt)]=="TAU"])

## [1] 3426.667

IQR(tt[Treat[names(tt)]!="TAU"])

## [1] 6958.201

sel1<- DFALL$fromStart>=10 & DFALL$fromStart<= 21& DFALL$Weekdays%in%c("Montag","Dienstag","Mittwoch","Donnerstag","Freitag")
tt<-tapply(DFALL$StepTotal[sel1],DFALL$PatientID[sel1],mean)
DF1<-data.frame(MeanStepsDay=tt,Treatment=Treat[names(tt)])
g1<-ggplot(DF1,aes(y=MeanStepsDay,x=Treatment) )+geom_boxplot() +labs(title="mean daily steps days 10-21")
print(g1)


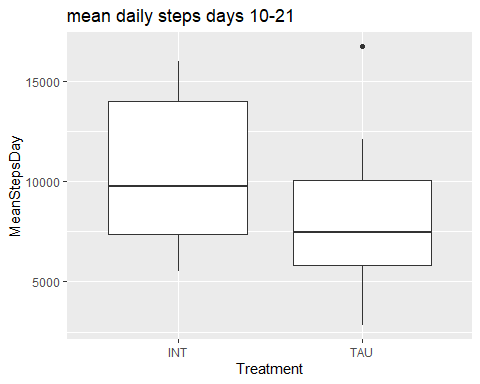


wilcox.test(tt[Treat[names(tt)]=="TAU"],tt[Treat[names(tt)]!="TAU"] )

##
## Wilcoxon rank sum exact test
##
## data: tt[Treat[names(tt)] == "TAU"] and tt[Treat[names(tt)] != "TAU"]
## W = 78, p-value = 0.1087
## alternative hypothesis: true location shift is not equal to 0

table(Treat[names(tt)]=="TAU")

##
## FALSE TRUE
## 14 17

median(tt[Treat[names(tt)]=="TAU"])

## [1] 7483.125

median(tt[Treat[names(tt)]!="TAU"])

## [1] 9760.375

IQR(tt[Treat[names(tt)]=="TAU"])

## [1] 4274.111

IQR(tt[Treat[names(tt)]!="TAU"])

## [1] 6612.833

# Split by Post Intervention Hamd17 evaluation

sel1<- DFALL$fromStart>=10 & DFALL$fromStart<=21 & DFALL$Weekdays%in%c("Montag","Dienstag","Mittwoch","Donnerstag","Freitag")
tt<-tapply(DFALL$StepTotal[sel1],DFALL$PatientID[sel1],mean)
DF1<-data.frame(MeanStepsDay=tt,Hamd17Group=Hamd17Group[names(tt)])
g1<-ggplot(DF1,aes(y=MeanStepsDay,x=Hamd17Group) )+geom_boxplot() +labs(title="mean daily steps days 10-21")
print(g1)


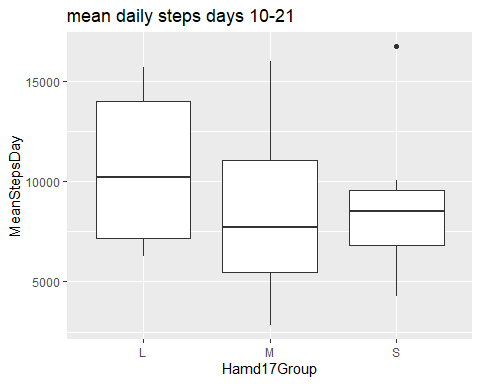


wilcox.test(tt[Hamd17Group[names(tt)]=="L"],tt[Hamd17Group[names(tt)]=="S"] )

##
## Wilcoxon rank sum exact test
##
## data: tt[Hamd17Group[names(tt)] == "L"] and tt[Hamd17Group[names(tt)] == "S"]
## W = 58, p-value = 0.3154
## alternative hypothesis: true location shift is not equal to 0

table(Hamd17Group[names(tt)])

##
## L M S
## 10 12 9

median(tt[Hamd17Group[names(tt)]=="L"]) #"Light"

## [1] 10201.06

median(tt[Hamd17Group[names(tt)]!="M"])

## [1] 9233.875

median(tt[Hamd17Group[names(tt)]!="S"]) #"heavy depressed"

## [1] 8636.812

# Hourly data

Function to read hour resolved step data:

readHSteps<-function(Patient,start,stop,restrict=TRUE){
 f<-list.files("./Daten_JK/",paste(Patient,"_hourlySteps",sep="",collapse = ""),full.names = T)
 StepsCSV<-read.csv(f[1])
 TimePoints<-parse_date_time(StepsCSV[,1],'%m/%d/%Y %I:%M:%S %p')
 Weeks<-week(TimePoints)
 Weeks<-Weeks-min(Weeks)
 WD<-weekdays(TimePoints)
 DF<-data.frame(ActivityHour=TimePoints,Weekdays=WD,week=Weeks,StepTotal=StepsCSV[,2],LastWeek=(TimePoints> dmy(E)-days(10)) )
 DF$toEnd<- -(dmy(stop)-date(TimePoints))
 DF$fromStart <- as.numeric(date(TimePoints)- dmy(start))
 DF$PatientID<-Patient
 if(restrict){
 selTime<- date(TimePoints) >=dmy(S) & date(TimePoints)<=dmy(E)
 DF[selTime,]
 }else DF[,]
}

Concatenate

for(i in 1:33){

 P<- Uebersicht[i,"Probandennummer"]
 if(P=="P510040") next
 S<- Uebersicht[i,"X1..EG"]
 E<-Uebersicht[i,"X4.EG..Ende"]
 DF<-readHSteps(P,S,E,T)

 if(nrow(DF)==0) next
 DF$Intervention<- Uebersicht$Intervention[i]
 #DF$PatientID<-P
 DF$Post.HAMD<-Uebersicht$Post.HAMD17[i]
 DF$Prä.HAMD<-Uebersicht$Prä.HAMD17[i]
 DF$Post.BDI<-Uebersicht$Post.BDI[i]
 DF$Prä.BDI<-Uebersicht$Prä.BDI[i]
 DF$Post.HAMD.GROUP<-"M"
 if (Uebersicht$Post.HAMD17[i]<= 11) DF$Post.HAMD.GROUP<-"L"
 if (Uebersicht$Post.HAMD17[i]>= 21) DF$Post.HAMD.GROUP<-"S"
 DF$Prä.HAMD.GROUP<-"M"
 if (Uebersicht$Prä.HAMD17[i]<= 11) DF$Prä.HAMD.GROUP<-"L"
 if (Uebersicht$Prä.HAMD17[i]>= 21) DF$Prä.HAMD.GROUP<-"S"


 if(i ==1) DFALL<-DF else DFALL<-rbind(DFALL,DF)
}


DFAll_H_Steps<-DFALL

# Plot

sel<-DFAll_H_Steps$Weekdays%in%c("Montag","Dienstag","Mittwoch","Donnerstag","Freitag")&hour(DFAll_H_Steps$ActivityHour)%in%c(7:21)& DFAll_H_Steps$StepTotal>0 & DFAll_H_Steps$fromStart<=21
DFAll_H_Steps1<-subset(DFAll_H_Steps,sel)
patient_day<-paste(DFAll_H_Steps1$PatientID,DFAll_H_Steps1$toEnd,sep="_")

DF_H_week_day<-data.frame(MedSteps=tapply(DFAll_H_Steps1$StepTotal,patient_day,median),
 toEnd=tapply(DFAll_H_Steps1$toEnd,patient_day,function(v)v[1]),
 PatientID= tapply(DFAll_H_Steps1$PatientID,patient_day,function(v)v[1])
 )
#DF_H_week_afternoon<-data.frame(MedSteps=tapply(DFAll_H_Steps1$StepTotal,patient_day,mean),toEnd=tapply(DFAll_H_Steps1$toEnd,patient_day,function(v)v[1]), PatientID= tapply(DFAll_H_Steps1$PatientID,patient_day,function(v)v[1]) )
DF_H_week_day$Post.HAMD.GROUP<- tapply(DFAll_H_Steps1$Post.HAMD.GROUP,patient_day,function(v)v[1])
DF_H_week_day$fromStart<- tapply(DFAll_H_Steps1$fromStart,patient_day,function(v)v[1])

#gg1<- ggplot(DF_H_week_afternoon,aes(fromStart,MedSteps ,color=Post.HAMD.GROUP))+geom_line(aes(group=PatientID,colour=Post.HAMD.GROUP ))+geom_smooth(method="loess",aes(group=Post.HAMD.GROUP,color=Post.HAMD.GROUP))
gg1<- ggplot(DF_H_week_day,aes(fromStart,MedSteps ,color=Post.HAMD.GROUP))+geom_smooth(method="loess",aes(group=Post.HAMD.GROUP,color=Post.HAMD.GROUP))
print(gg1)

## `geom_smooth()` using formula 'y ~ x'


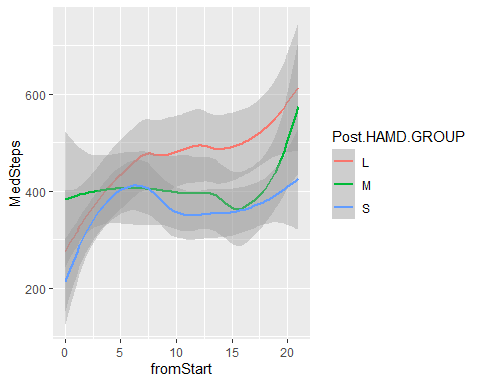
 # by Treatment

sel<-DFAll_H_Steps$Weekdays%in%c("Montag","Dienstag","Mittwoch","Donnerstag","Freitag")&hour(DFAll_H_Steps$ActivityHour)%in%c(7:21)& DFAll_H_Steps$StepTotal>0 & DFAll_H_Steps$fromStart<=21
DFAll_H_Steps1<-subset(DFAll_H_Steps,sel)
patient_day<-paste(DFAll_H_Steps1$PatientID,DFAll_H_Steps1$toEnd,sep="_")

DF_H_week_day<-data.frame(MedSteps=tapply(DFAll_H_Steps1$StepTotal,patient_day,median),
 toEnd=tapply(DFAll_H_Steps1$toEnd,patient_day,function(v)v[1]),
 PatientID= tapply(DFAll_H_Steps1$PatientID,patient_day,function(v)v[1])
 )
DF_H_week_day$fromStart<- tapply(DFAll_H_Steps1$fromStart,patient_day,function(v)v[1])

DF_H_week_day$Intervention<- Treat[DF_H_week_day$PatientID]
gg1<- ggplot(DF_H_week_day,aes(fromStart,MedSteps ,color=Intervention))+geom_smooth(method="loess",aes(group=Intervention,color=Intervention))
print(gg1)

## `geom_smooth()` using formula 'y ~ x'


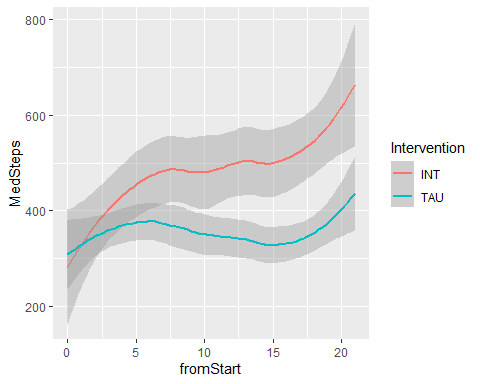


(table(DF_H_week_day$PatientID)) # Two patients missing

##
## P510001 P510002 P510005 P510006 P510007 P510008 P510009 P510011 P510014 P510017
## 7 15 11 16 15 14 14 15 14 14
## P510018 P510021 P510022 P510023 P510024 P510025 P510026 P510030 P510032 P510033
## 15 16 16 11 12 16 15 15 16 16
## P510036 P510037 P510038 P510039 P510041 P510042 P510043 P510044 P510045 P510046
## 16 15 13 15 16 16 14 14 14 12
## P510047
## 16

# Summaries

sel<-DFAll_H_Steps$Weekdays%in%c("Montag","Dienstag","Mittwoch","Donnerstag","Freitag")&hour(DFAll_H_Steps$ActivityHour)%in%c(7:21)& DFAll_H_Steps$StepTotal>=0 & DFAll_H_Steps$fromStart<=21 & DFAll_H_Steps$fromStart>=10
DFAll_H_Steps1<-subset(DFAll_H_Steps,sel)
tt<-tapply(DFAll_H_Steps1$StepTotal,DFAll_H_Steps1$PatientID,median)

DF_box<-data.frame(H_steps_median=tt,PatientID=names(tt),Intervention=Treat[names(tt)] )
gg1<-ggplot(DF_box,aes(y=H_steps_median,x=Intervention))+geom_boxplot()+labs(title =" TAU vs INT median steps day 10-21")
print(gg1)


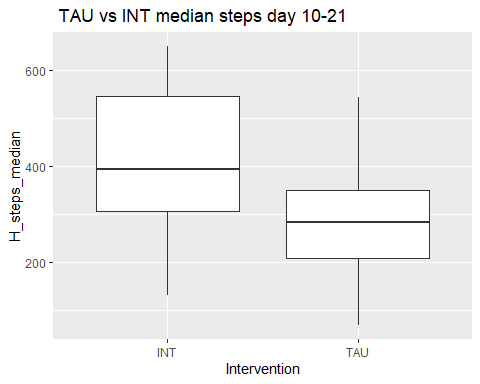


wilcox.test(DF_box$H_steps_median[DF_box$Intervention=="TAU"],DF_box$H_steps_median[DF_box$Intervention!="TAU"] )

##
## Wilcoxon rank sum exact test
##
## data: DF_box$H_steps_median[DF_box$Intervention == "TAU"] and DF_box$H_steps_median[DF_box$Intervention != "TAU"]
## W = 56, p-value = 0.01157
## alternative hypothesis: true location shift is not equal to 0

#t.test(DF_box$H_steps_median[DF_box$Intervention=="TAU"],DF_box$H_steps_median[DF_box$Intervention!="TAU"] )
print(paste("global median(IQR): ",median(DF_box$H_steps_median[]),"(",IQR(DF_box$H_steps_median[]),")"))

## [1] "global median(IQR): 316 ( 203.25 )"

print(paste("TAU median(IQR):", median(DF_box$H_steps_median[DF_box$Intervention=="TAU"]),"(",IQR(DF_box$H_steps_median[DF_box$Intervention=="TAU"]),")"))

## [1] "TAU median(IQR): 283 ( 141 )"

print(paste("Intervention median(IQR)",median(DF_box$H_steps_median[DF_box$Intervention!="TAU"]),"(",IQR(DF_box$H_steps_median[DF_box$Intervention!="TAU"]),")"))

## [1] "Intervention median(IQR) 394.5 ( 239.125 )"

table(DF_box$Intervention=="TAU")

##
## FALSE TRUE
## 14 17

by Post-treatment Hamd17

sel<-DFAll_H_Steps$Weekdays%in%c("Montag","Dienstag","Mittwoch","Donnerstag","Freitag")&hour(DFAll_H_Steps$ActivityHour)%in%c(7:21)& DFAll_H_Steps$StepTotal>=0 & DFAll_H_Steps$fromStart<=21 & DFAll_H_Steps$fromStart>=10
DFAll_H_Steps1<-subset(DFAll_H_Steps,sel)
tt<-tapply(DFAll_H_Steps1$StepTotal,DFAll_H_Steps1$PatientID,median)

DF_box<-data.frame(H_steps_median=tt,PatientID=names(tt),Post.HAMD.GROUP=Hamd17Group[names(tt)] )
gg1<-ggplot(DF_box,aes(y=H_steps_median,x=Post.HAMD.GROUP))+geom_boxplot()+labs(title =" TAU vs INT median steps day 10-21")
print(gg1)


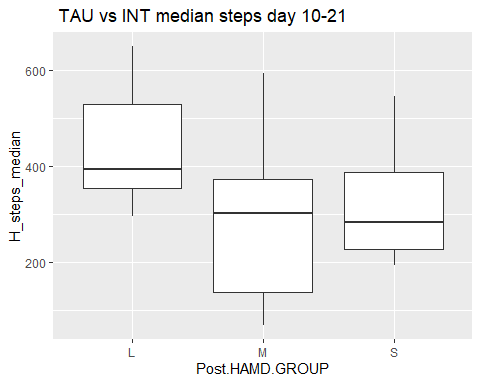


wilcox.test(DF_box$H_steps_median[DF_box$Post.HAMD.GROUP=="L"],DF_box$H_steps_median[DF_box$Post.HAMD.GROUP=="S"] )

##
## Wilcoxon rank sum exact test
##
## data: DF_box$H_steps_median[DF_box$Post.HAMD.GROUP == "L"] and DF_box$H_steps_median[DF_box$Post.HAMD.GROUP == "S"]
## W = 70, p-value = 0.04347
## alternative hypothesis: true location shift is not equal to 0

median(DF_box$H_steps_median[DF_box$Post.HAMD.GROUP=="L"])

## [1] 394.5

median(DF_box$H_steps_median[DF_box$Post.HAMD.GROUP=="S"])

## [1] 283

IQR(DF_box$H_steps_median[DF_box$Post.HAMD.GROUP=="L"])

## [1] 174.375

IQR(DF_box$H_steps_median[DF_box$Post.HAMD.GROUP=="S"])

## [1] 160

# SLEEP

## Individual daily summary data Sleep

readDaylySleep<-function(Patient,start,stop){
 f<-list.files("./Daten_JK/",pattern = paste(Patient,"_sleepStagesDay_",sep="",collapse = ""),full.names = T)
 #dmy("20.08.2020")
 #print(f)
 print(f)
 StepsCSV<-read.csv(f[1])
 TimePoints<-parse_date_time(StepsCSV[,1],'%m/%d/%Y %I:%M:%S %p')
 StepsCSV["TimePoints"]<-TimePoints
 StepsCSV[,"Weekdays"] <- weekdays(TimePoints)
 selTime<- TimePoints >=dmy(start) & TimePoints<=dmy(stop)
 StepsCSV$fromStart<-as.numeric(date(TimePoints)-dmy(start))
 StepsCSV$toEnd<-as.numeric(date(TimePoints)-dmy(stop))
 StepsCSV$PatientID<-Patient
 StepsCSV[selTime,]
}

We create a long Data-frame containing all patient data by concatenating all individual files. For reasons of interpretation only days with one single sleep record are retained.

for(i in 1:33){

 P<- Uebersicht[i,"Probandennummer"]
 if(P=="P510040") next
 S<- Uebersicht[i,"X1..EG"]
 E<-Uebersicht[i,"X4.EG..Ende"]
 DF<-readDaylySleep(P,S,E)
 if(nrow(DF)==0) next
 DF$Intervention<- Uebersicht$Intervention[i]
 DF$Post.HAMD<-Uebersicht$Post.HAMD17[i]
 DF$Prä.HAMD<-Uebersicht$Prä.HAMD17[i]
 DF$Post.BDI<-Uebersicht$Post.BDI[i]
 DF$Prä.BDI<-Uebersicht$Prä.BDI[i]
 DF$Post.HAMD.GROUP<-"M"
 if (Uebersicht$Post.HAMD17[i]<= 11) DF$Post.HAMD.GROUP<-"L"
 if (Uebersicht$Post.HAMD17[i]>= 21) DF$Post.HAMD.GROUP<-"S"
 DF$Prä.HAMD.GROUP<-"M"
 if (Uebersicht$Prä.HAMD17[i]<= 11) DF$Prä.HAMD.GROUP<-"L"
 if (Uebersicht$Prä.HAMD17[i]>= 21) DF$Prä.HAMD.GROUP<-"S"
 if(i ==1) DFALL<-DF else DFALL<-rbind(DFALL,DF)
}

## [1] "./Daten_JK/P510022_sleepStagesDay_20191020_20210526.csv"
## [1] "./Daten_JK/P510023_sleepStagesDay_20191020_20210526.csv"
## [1] "./Daten_JK/P510024_sleepStagesDay_20191020_20210526.csv"
## [1] "./Daten_JK/P510032_sleepStagesDay_20191020_20210526.csv"
## [1] "./Daten_JK/P510033_sleepStagesDay_20191020_20210526.csv"
## [1] "./Daten_JK/P510036_sleepStagesDay_20191020_20210526.csv"
## [1] "./Daten_JK/P510042_sleepStagesDay_20191020_20210526.csv"
## [1] "./Daten_JK/P510001_sleepStagesDay_20191020_20210526.csv"
## [1] "./Daten_JK/P510002_sleepStagesDay_20191020_20210526.csv"
## [1] "./Daten_JK/P510007_sleepStagesDay_20191020_20210526.csv"
## [1] "./Daten_JK/P510011_sleepStagesDay_20191020_20210526.csv"
## [1] "./Daten_JK/P510014_sleepStagesDay_20191020_20210526.csv"
## [1] "./Daten_JK/P510017_sleepStagesDay_20191020_20210526.csv"
## [1] "./Daten_JK/P510018_sleepStagesDay_20191020_20210526.csv"
## [1] "./Daten_JK/P510021_sleepStagesDay_20191020_20210526.csv"
## [1] "./Daten_JK/P510025_sleepStagesDay_20191020_20210526.csv"
## [1] "./Daten_JK/P510026_sleepStagesDay_20191020_20210526.csv"
## [1] "./Daten_JK/P510030_sleepStagesDay_20191020_20210526.csv"
## [1] "./Daten_JK/P510041_sleepStagesDay_20191020_20210526.csv"
## [1] "./Daten_JK/P510043_sleepStagesDay_20191020_20210526.csv"
## [1] "./Daten_JK/P510044_sleepStagesDay_20191020_20210526.csv"
## [1] "./Daten_JK/P510045_sleepStagesDay_20191020_20210526.csv"
## [1] "./Daten_JK/P510046_sleepStagesDay_20191020_20210526.csv"
## [1] "./Daten_JK/P510047_sleepStagesDay_20191020_20210526.csv"
## [1] "./Daten_JK/P510005_sleepStagesDay_20191020_20210526.csv"
## [1] "./Daten_JK/P510006_sleepStagesDay_20191020_20210526.csv"
## [1] "./Daten_JK/P510008_sleepStagesDay_20191020_20210526.csv"
## [1] "./Daten_JK/P510009_sleepStagesDay_20191020_20210526.csv"
## [1] "./Daten_JK/P510020_sleepStagesDay_20191020_20210526.csv"
## [1] "./Daten_JK/P510037_sleepStagesDay_20191020_20210526.csv"
## [1] "./Daten_JK/P510038_sleepStagesDay_20191020_20210526.csv"
## [1] "./Daten_JK/P510039_sleepStagesDay_20191020_20210526.csv"

#
DFALLSleep<-DFALL
DFALLSleep<- DFALLSleep[DFALLSleep$TotalSleepRecords==1,]

hist(table(DFALLSleep$PatientID))


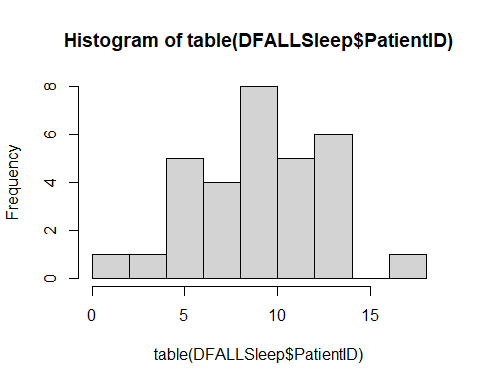


median(table(DFALLSleep$PatientID))

## [1] 10

The median number of usable sleep records is <10 indicting problematic data. Plots dont show usable trends. #

gg1<-ggplot(DFALLSleep,aes(fromStart,TotalMinutesAsleep,color=Intervention))+geom_line(aes(group=PatientID,colour=Intervention ))+geom_smooth(method="loess",aes(group=Intervention,color=Intervention))
print(gg1)

## `geom_smooth()` using formula 'y ~ x'


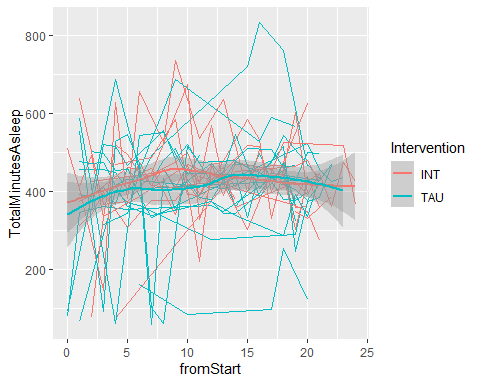


## Individual high resolution data: Sleep

Individual data sets are read through accessory functions which take patientID, start and a stop dates.

read30sSleep<-function(patient,S,E){
 f<-list.files("./Daten_JK/",paste(patient,"_30secondSleepStages",sep="",collapse = ""),full.names = T)
 SleepCSV<-read.csv(f[1])
 TimePoints<-parse_date_time(SleepCSV[,2],'%m/%d/%Y %I:%M:%S %p')
 Weeks<-isoweek(TimePoints)
 Weeks<-Weeks-min(Weeks)
 WD<-weekdays(TimePoints)
 DF<-data.frame(LogId=SleepCSV$LogId,LogIdN=as.numeric(factor(SleepCSV$LogId)),Time=TimePoints,Weekdays=WD,week=Weeks,SleepLevel=(factor(SleepCSV$Level)),LastWeek=(TimePoints> dmy(E)-days(10)) )
 selTime<- TimePoints >=dmy(S) & TimePoints<=dmy(E)

 DF[selTime,]

}

For instance we can access and store the 30s resolved of the patient “P510033” who started “16.11.2020” and ended “16.11.2020” by running:

i=5 # "P510033
P= Uebersicht[i,"Probandennummer"] # "P510033"
S<- Uebersicht[i,"X1..EG"] #"16.11.2020"
E<-Uebersicht[i,"X4.EG..Ende"] #"16.11.2020"
DF<-read30sSleep(P,S =S ,E = E)
head(DF)

## LogId LogIdN Time Weekdays week SleepLevel LastWeek
## 1 29800960094 1 2020-11-19 00:02:30 Donnerstag 0 wake FALSE
## 2 29800960094 1 2020-11-19 00:03:00 Donnerstag 0 light FALSE
## 3 29800960094 1 2020-11-19 00:03:30 Donnerstag 0 light FALSE
## 4 29800960094 1 2020-11-19 00:04:00 Donnerstag 0 light FALSE
## 5 29800960094 1 2020-11-19 00:04:30 Donnerstag 0 light FALSE
## 6 29800960094 1 2020-11-19 00:05:00 Donnerstag 0 light FALSE

An individual record can be inspected.

recordID=1 # the maximal available number of records max(DF$LogIdN)
startRecord<- min(DF$Time[DF$LogIdN==recordID])
endRecord<- max(DF$Time[DF$LogIdN==recordID])
tt<-(DF$Time[DF$LogIdN==recordID][]-startRecord)/60 #
DFP<-data.frame(time=tt,level=as.numeric((DF$SleepLevel[DF$LogIdN==recordID])))
gg<-ggplot(DFP,aes(x=time,y=level))+geom_step()+scale_y_continuous(name = "Level",labels = c("wake","rem","light","deep"),breaks=c(4,3,2,1))+labs(title=paste("ID:",P), subtitle = paste(startRecord,"---", endRecord ))
print(gg)

## Don't know how to automatically pick scale for object of type difftime. Defaulting to continuous.


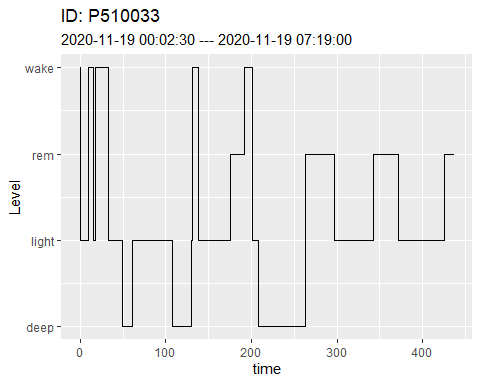


# deriving additional Information and creating long format data.frames

Individual Data is used to derive additional information

getNightStats<-function(DF,S,E){

LogIds<-levels((factor(DF$LogId)))
print(LogIds)
DFR<-data.frame(remstart=numeric(),rem1=numeric(), wakeT=numeric(),date=as.Date(character()),Weekday=character(),week=numeric(),LastWeek=logical(),toEnd=numeric(),fromStart=numeric())
for(j in LogIds){
 print(j)
 sel<-DF$LogId==j
 Levels30s<-as.character(DF$SleepLevel[sel] )
 v<-rle(as.character(Levels30s))
 t_0<-which(v$value=="rem")[1]
 if(is.na(t_0)) next
 t_rem1<-sum(v$lengths[1:(t_0-1)])
 day<- date(DF$Time[sel][1]-hours(5))
 w<-DF$week[sel][1]
 wday<-weekdays(day)
 DFR[j,1]=t_rem1
 DFR[j,2]=v$lengths[t_0]
 DFR[j,3]=sum(v$lengths[v$values=="wake"])
 DFR[j,4]=day
 DFR[j,5]<-wday
 DFR[j,6]<-w
 DFR[j,7]<-DF$LastWeek[sel][1]
 DFR[j,8] <- -(dmy(E)-day)
 DFR[j,9] <- day-dmy(S)

}
DFR
}

for(i in 1:33){

 P<- Uebersicht[i,"Probandennummer"]
 if(P=="P510040") next
 S<- Uebersicht[i,"X1..EG"]
 E<-Uebersicht[i,"X4.EG..Ende"]
 DF1<-read30sSleep(P,S,E)
 DF<-getNightStats(DF1[,],S,E)
 if(nrow(DF)==0) next
 DF$PatientID<-P
 DF$Post.HAMD<-Uebersicht$Post.HAMD17[i]
 DF$Prä.HAMD<-Uebersicht$Prä.HAMD17[i]
 DF$Post.BDI<-Uebersicht$Post.BDI[i]
 DF$Prä.BDI<-Uebersicht$Prä.BDI[i]
 DF$Post.HAMD.GROUP<-"M"
 if (Uebersicht$Post.HAMD17[i]<= 11) DF$Post.HAMD.GROUP<-"L"
 if (Uebersicht$Post.HAMD17[i]>= 21) DF$Post.HAMD.GROUP<-"S"
 DF$Prä.HAMD.GROUP<-"M"
 if (Uebersicht$Prä.HAMD17[i]<= 11) DF$Prä.HAMD.GROUP<-"L"
 if (Uebersicht$Prä.HAMD17[i]>= 21) DF$Prä.HAMD.GROUP<-"S"


 if(i ==1) DFALL<-DF else DFALL<-rbind(DFALL,DF)
}

## [1] "28725452296" "28725452299" "28725452300" "28815137450" "28815137451"
## [1] "28725452296"
## [1] "28725452299"
## [1] "28725452300"
## [1] "28815137450"
## [1] "28815137451"
## [1] "28542591122" "28542591123" "28607467143" "28607467144" "28607467145"
## [6] "28705488454" "28705488455" "28705488456" "28705488459"
## [1] "28542591122"
## [1] "28542591123"
## [1] "28607467143"
## [1] "28607467144"
## [1] "28607467145"
## [1] "28705488454"
## [1] "28705488455"
## [1] "28705488456"
## [1] "28705488459"

## Warning in min(Weeks): kein nicht-fehlendes Argument für min; gebe Inf zurück

## character(0)
## [1] "29734730120" "29824911661"
## [1] "29734730120"
## [1] "29824911661"
## [1] "29800960094" "29800960096" "29800960098" "29887496916" "29887496918"
## [6] "29907036115" "29934580146"
## [1] "29800960094"
## [1] "29800960096"
## [1] "29800960098"
## [1] "29887496916"
## [1] "29887496918"
## [1] "29907036115"
## [1] "29934580146"
## [1] "29996915928" "29996915929" "29996915930" "29996915933" "29996915935"
## [6] "30024341367" "30093874116" "30179091360" "30179091361"
## [1] "29996915928"
## [1] "29996915929"
## [1] "29996915930"
## [1] "29996915933"
## [1] "29996915935"
## [1] "30024341367"
## [1] "30093874116"
## [1] "30179091360"
## [1] "30179091361"
## [1] "31469611109" "31469611113" "31561545457" "31561545462" "31561545464"
## [6] "31561545466" "31676346055" "31676346058" "31676346059" "31676346060"
## [1] "31469611109"
## [1] "31469611113"
## [1] "31561545457"
## [1] "31561545462"
## [1] "31561545464"
## [1] "31561545466"
## [1] "31676346055"
## [1] "31676346058"
## [1] "31676346059"
## [1] "31676346060"
## [1] "24707148769" "24707148770" "24707148771" "24707148776" "24707148777"
## [6] "24707148779" "24707148780"
## [1] "24707148769"
## [1] "24707148770"
## [1] "24707148771"
## [1] "24707148776"
## [1] "24707148777"
## [1] "24707148779"
## [1] "24707148780"
## [1] "24511238918" "24511238919" "24511238921" "24511238922" "24511238923"
## [6] "24707163952" "24707163954" "24707163955" "24707163956"
## [1] "24511238918"
## [1] "24511238919"
## [1] "24511238921"
## [1] "24511238922"
## [1] "24511238923"
## [1] "24707163952"
## [1] "24707163954"
## [1] "24707163955"
## [1] "24707163956"
## [1] "25941166424" "25941166425" "25941166426" "25941166427" "25941166428"
## [6] "25941166429" "25941166430" "26176112958" "26176112959" "26176112960"
## [11] "26176112962"
## [1] "25941166424"
## [1] "25941166425"
## [1] "25941166426"
## [1] "25941166427"
## [1] "25941166428"
## [1] "25941166429"
## [1] "25941166430"
## [1] "26176112958"
## [1] "26176112959"
## [1] "26176112960"
## [1] "26176112962"
## [1] "27148842781" "27148842782" "27148842783" "27148842784" "27148842785"
## [6] "27367352219" "27367352220" "27367352221" "27367352222" "27367352223"
## [11] "27367352224" "27367352225" "27396272412"
## [1] "27148842781"
## [1] "27148842782"
## [1] "27148842783"
## [1] "27148842784"
## [1] "27148842785"
## [1] "27367352219"
## [1] "27367352220"
## [1] "27367352221"
## [1] "27367352222"
## [1] "27367352223"
## [1] "27367352224"
## [1] "27367352225"
## [1] "27396272412"
## [1] "27972513803" "27972513804" "27972513805" "27972513806" "27972513808"
## [6] "27972513810"
## [1] "27972513803"
## [1] "27972513804"
## [1] "27972513805"
## [1] "27972513806"
## [1] "27972513808"
## [1] "27972513810"
## [1] "28070226781" "28070226782" "28070226784" "28070226785" "28070226787"
## [6] "28162844811" "28162844813" "28162844814" "28162844815" "28227771644"
## [11] "28227771650" "28227771652" "28227771653"
## [1] "28070226781"
## [1] "28070226782"
## [1] "28070226784"
## [1] "28070226785"
## [1] "28070226787"
## [1] "28162844811"
## [1] "28162844813"
## [1] "28162844814"
## [1] "28162844815"
## [1] "28227771644"
## [1] "28227771650"
## [1] "28227771652"
## [1] "28227771653"
## [1] "28069852356" "28069852357" "28135494965" "28135494967" "28254202411"
## [6] "28254202413" "28254202415" "28254202416"
## [1] "28069852356"
## [1] "28069852357"
## [1] "28135494965"
## [1] "28135494967"
## [1] "28254202411"
## [1] "28254202413"
## [1] "28254202415"
## [1] "28254202416"
## [1] "28486462507" "28486462508" "28486462510" "28486462511" "28605453388"
## [6] "28605453389" "28605453390" "28605453391" "28636551984" "28636551986"
## [1] "28486462507"
## [1] "28486462508"
## [1] "28486462510"
## [1] "28486462511"
## [1] "28605453388"
## [1] "28605453389"
## [1] "28605453390"
## [1] "28605453391"
## [1] "28636551984"
## [1] "28636551986"
## [1] "28636979797" "28720237288" "28720237292" "28831689384" "28831689386"
## [1] "28636979797"
## [1] "28720237288"
## [1] "28720237292"
## [1] "28831689384"
## [1] "28831689386"
## [1] "28635139237" "28635139239" "28635139240" "28635139241" "28635139242"
## [6] "28635139243" "28635139244" "28720257294" "28814636472" "28814636473"
## [11] "28814636478" "28814636482" "28814636486"
## [1] "28635139237"
## [1] "28635139239"
## [1] "28635139240"
## [1] "28635139241"
## [1] "28635139242"
## [1] "28635139243"
## [1] "28635139244"
## [1] "28720257294"
## [1] "28814636472"
## [1] "28814636473"
## [1] "28814636478"
## [1] "28814636482"
## [1] "28814636486"
## [1] "29285296236" "29285296240" "29285296241" "29387404108" "29387404109"
## [6] "29387404110" "29387404111"
## [1] "29285296236"
## [1] "29285296240"
## [1] "29285296241"
## [1] "29387404108"
## [1] "29387404109"
## [1] "29387404110"
## [1] "29387404111"
## [1] "31455817675" "31455817676" "31548426163" "31548426164" "31548426165"
## [6] "31548426166" "31548426167" "31548426170" "31645696968" "31645696969"
## [1] "31455817675"
## [1] "31455817676"
## [1] "31548426163"
## [1] "31548426164"
## [1] "31548426165"
## [1] "31548426166"
## [1] "31548426167"
## [1] "31548426170"
## [1] "31645696968"
## [1] "31645696969"
## [1] "31564357729" "31676463252" "31676463253" "31676463256" "31748664588"
## [6] "31748664589"
## [1] "31564357729"
## [1] "31676463252"
## [1] "31676463253"
## [1] "31676463256"
## [1] "31748664588"
## [1] "31748664589"
## [1] "31763666930" "31938826981" "31938826983"
## [1] "31763666930"
## [1] "31938826981"
## [1] "31938826983"
## [1] "31765274630" "31765274635" "31847714641" "31847714645" "31939818481"
## [6] "31939818485" "31939818486" "31939818487" "31939818488"
## [1] "31765274630"
## [1] "31765274635"
## [1] "31847714641"
## [1] "31847714645"
## [1] "31939818481"
## [1] "31939818485"
## [1] "31939818486"
## [1] "31939818487"
## [1] "31939818488"
## [1] "31847322301" "31847322302" "31847322303"
## [1] "31847322301"
## [1] "31847322302"
## [1] "31847322303"
## [1] "31816999134" "31816999135" "31816999136" "31816999138" "31913762553"
## [6] "31913762554" "31913762556" "32051399188" "32051399191"
## [1] "31816999134"
## [1] "31816999135"
## [1] "31816999136"
## [1] "31816999138"
## [1] "31913762553"
## [1] "31913762554"
## [1] "31913762556"
## [1] "32051399188"
## [1] "32051399191"
## [1] "25419174306" "25419174308" "25419174309" "25419174311" "25419174312"
## [6] "25461706996" "25461706998"
## [1] "25419174306"
## [1] "25419174308"
## [1] "25419174309"
## [1] "25419174311"
## [1] "25419174312"
## [1] "25461706996"
## [1] "25461706998"
## [1] "25537883734" "25537883735" "25537883736" "25537883737" "25537883738"
## [6] "25537883739" "25640233267" "25640233269" "25640233270" "25640233271"
## [11] "25640233272" "25640233273" "25640233274" "25783604120" "25783604121"
## [16] "25783604123" "25783604124" "25783604125"
## [1] "25537883734"
## [1] "25537883735"
## [1] "25537883736"
## [1] "25537883737"
## [1] "25537883738"
## [1] "25537883739"
## [1] "25640233267"
## [1] "25640233269"
## [1] "25640233270"
## [1] "25640233271"
## [1] "25640233272"
## [1] "25640233273"
## [1] "25640233274"
## [1] "25783604120"
## [1] "25783604121"
## [1] "25783604123"
## [1] "25783604124"
## [1] "25783604125"
## [1] "26148015502" "26148015505" "26148015506" "26148015507" "26148015508"
## [6] "26148015509" "26148015510" "26311588591" "26311588592" "26311588594"
## [11] "26311588595" "26311588597" "26311588598"
## [1] "26148015502"
## [1] "26148015505"
## [1] "26148015506"
## [1] "26148015507"
## [1] "26148015508"
## [1] "26148015509"
## [1] "26148015510"
## [1] "26311588591"
## [1] "26311588592"
## [1] "26311588594"
## [1] "26311588595"
## [1] "26311588597"
## [1] "26311588598"
## [1] "26148253435" "26310387085" "26310387089" "26310387090" "26310387091"
## [6] "26310387093"
## [1] "26148253435"
## [1] "26310387085"
## [1] "26310387089"
## [1] "26310387090"
## [1] "26310387091"
## [1] "26310387093"

## Warning in min(Weeks): kein nicht-fehlendes Argument für min; gebe Inf zurück

## character(0)
## [1] "30433560918" "30495625598" "30565736107" "30565736110" "30598796922"
## [6] "30670515776" "30670515779"
## [1] "30433560918"
## [1] "30495625598"
## [1] "30565736107"
## [1] "30565736110"
## [1] "30598796922"
## [1] "30670515776"
## [1] "30670515779"
## [1] "30493748804" "30493748805" "30542472524" "30643407747" "30643407748"
## [6] "30643407751" "30643407755" "30643407756" "30745001263"
## [1] "30493748804"
## [1] "30493748805"
## [1] "30542472524"
## [1] "30643407747"
## [1] "30643407748"
## [1] "30643407751"
## [1] "30643407755"
## [1] "30643407756"
## [1] "30745001263"
## [1] "30977017350" "30977017351" "30977017352" "31062459470" "31062459475"
## [6] "31062459476" "31062459477" "31132709160" "31132709161" "31179819292"
## [1] "30977017350"
## [1] "30977017351"
## [1] "30977017352"
## [1] "31062459470"
## [1] "31062459475"
## [1] "31062459476"
## [1] "31062459477"
## [1] "31132709160"
## [1] "31132709161"
## [1] "31179819292"

DFALLSleep30s<-DFALL

Similar to the daily summaries there are many missing records in the sleep data. (Potentially fixable errors dont add much)

t1<-table(DFALLSleep30s$PatientID)
print(t1)

##
## P510001 P510002 P510005 P510006 P510007 P510008 P510009 P510011 P510014 P510017
## 6 8 6 18 11 13 6 13 6 13
## P510018 P510021 P510022 P510023 P510025 P510026 P510030 P510032 P510033 P510036
## 8 9 5 9 4 12 6 1 7 9
## P510037 P510038 P510039 P510041 P510042 P510043 P510044 P510045 P510046 P510047
## 6 9 9 9 9 6 3 9 3 8

mean(t1)

## [1] 8.033333
